# Supplementary material for: Social influences in the experience of transition to or from long-term (chronic) pain: A systematic review of qualitative research studies
Source: PLoS One. 2025 Jul 10;20(7):e0327984. doi: 10.1371/journal.pone.0327984 (PMC12244478; doi:10.1371/journal.pone.0327984)
Supplement: S1 File — (PDF) [file pone.0327984.s001.pdf]

Systematic review

*Fields that have an **asterisk (*)** next to them means that they **must be answered. Word limits** are provided for each section. You will be unable to submit the form if the word limits are exceeded for any section. Registrant means the person filling out the form.*

1. * Review title.

Give the title of the review in English

Social and cultural influences in the experience of transition to or from long-term (chronic) pain: a meta-synthesis of qualitative research studies.

2. Original language title.

For reviews in languages other than English, give the title in the original language. This will be displayed with the English language title.

3. * Anticipated or actual start date.

Give the date the systematic review started or is expected to start.

22/03/2022

4. * Anticipated completion date.

Give the date by which the review is expected to be completed.

31/12/2022

5. * Stage of review at time of this submission.

This field uses answers to initial screening questions. It cannot be edited until after registration.

Tick the boxes to show which review tasks have been started and which have been completed.

Update this field each time any amendments are made to a published record.

The review has not yet started: No

Review stage	Started	Completed
Preliminary searches	Yes	No
Piloting of the study selection process	Yes	No
Formal screening of search results against eligibility criteria	Yes	No
Data extraction	No	No
Risk of bias (quality) assessment	No	No
Data analysis	No	No

Provide any other relevant information about the stage of the review here.

6. * Named contact.

The named contact is the guarantor for the accuracy of the information in the register record. This may be any member of the review team.

Rachael Gooberman-Hill

Email salutation (e.g. "Dr Smith" or "Joanne") for correspondence:
Professor Gooberman-Hill

7. * Named contact email.

Give the electronic email address of the named contact.

r.gooberman-hill@bristol.ac.uk

8. Named contact address

PLEASE NOTE this information will be published in the PROSPERO record so please do not enter private information, i.e. personal home address

Give the full institutional/organisational postal address for the named contact.

Bristol Medical School, University of Bristol, Learning and Research Building, Level 1, Southmead Hospital, Bristol BS10 5NB

9. Named contact phone number.

Give the telephone number for the named contact, including international dialling code.

00000000

10. * Organisational affiliation of the review.

Full title of the organisational affiliations for this review and website address if available. This field may be completed as 'None' if the review is not affiliated to any organisation.

University of Bristol

Organisation web address:
https://www.bristol.ac.uk/

11. * Review team members and their organisational affiliations.

Give the personal details and the organisational affiliations of each member of the review team. Affiliation refers to groups or organisations to which review team members belong.
NOTE: email and country now MUST be entered for each person, unless you are amending a published record.

Professor Rachael Gooberman-Hill. University of Bristol
Dr Elaine Wainwright. University of Aberdeen
Dr Amber Guest. University of Aberdeen
Dr Anica Zeyen. Royal Holloway University of London
Dr Hannah Sallis. University of Bristol

12. * Funding sources/sponsors.

Details of the individuals, organizations, groups, companies or other legal entities who have funded or sponsored the review.

MRC Advanced Pain Discovery Platform

Grant number(s)
State the funder, grant or award number and the date of award

MRC Advanced Pain Discovery Platform, MR/W004151/1, 2022-2025

13. * Conflicts of interest.

List actual or perceived conflicts of interest (financial or academic).

None

14. Collaborators.

Give the name and affiliation of any individuals or organisations who are working on the review but who are not listed as review team members. **NOTE: email and country must be completed for each person, unless you are amending a published record.**

15. * Review question.

State the review question(s) clearly and precisely. It may be appropriate to break very broad questions down into a series of related more specific questions. Questions may be framed or refined using PI(E)COS or similar where relevant.

The review will identify and characterise social and cultural influences in the experience of people (age 18+) who transition to or from chronic pain.

We are considering pain though the International Association for the Study of Pain (IASP) list of pain terms.

International Association for the Study of Pain (IASP) (1979). Pain terms: a list with definitions and notes on usage: recommended by the IASP Subcommittee on Taxonomy. Pain; 6:249.

16. * Searches.

State the sources that will be searched (e.g. Medline). Give the search dates, and any restrictions (e.g. language or publication date). Do NOT enter the full search strategy (it may be provided as a link or attachment below.)

A search of MEDLINE/PubMed, PsycINFO, EMBASE, CINAHL, Sociological Abstracts, Sociology Database, Social Sciences Citation Index and Web of Science, Scopus, Business Source Complete.

Search terms to include: chronic pain, occupation, work, employment, entrepreneur, environment, social, cultural, community, organisation(al), context.

Inclusion criteria:

1. Studies published from 1979 - 2022
2. Studies using empirical qualitative methods including interviews, focus groups, ethnographic or other qualitative methods of data collection, and reviews of qualitative studies
3. Qualitative studies focused on experience of transition to or from chronic pain
4. Studies including people aged 18 years and over
5. Studies in any language for which that translation support is available

Exclusion criteria:

1. Quantitative studies in which the main method of data collection is a survey in which participants were asked to provide ‘free text’ responses to one or several survey questions only.

Although we will carry out a search using the pre-specified approach, we will include further strategies if it becomes apparent during the work that doing so would be likely to yield further relevant studies. For instance, the study team will also check the reference lists of studies that the research team identify as key studies and include studies that are relevant and that have not been identified through our other approaches.

17. URL to search strategy.

Upload a file with your search strategy, or an example of a search strategy for a specific database, (including the keywords) in pdf or word format. In doing so you are consenting to the file being made publicly accessible.

Or provide a URL or link to the strategy. Do NOT provide links to your search **results**.

Yes I give permission for this file to be made publicly available

18. * Condition or domain being studied.

Give a short description of the disease, condition or healthcare domain being studied in your systematic review.

Chronic pain from any cause including pain that is improved or worsened.

19. * Participants/population.

Specify the participants or populations being studied in the review. The preferred format includes details of both inclusion and exclusion criteria.

People aged 18 years and over who have transitioned into or out of chronic pain.

20. * Intervention(s), exposure(s).

Give full and clear descriptions or definitions of the interventions or the exposures to be reviewed. The preferred format includes details of both inclusion and exclusion criteria.

21. * Comparator(s)/control.

Where relevant, give details of the alternatives against which the intervention/exposure will be compared (e.g. another intervention or a non-exposed control group). The preferred format includes details of both inclusion and exclusion criteria.

No intervention. Experiential accounts of their social and cultural influences on their experience of transitioning from or out of chronic pain.

22. * Types of study to be included.

Give details of the study designs (e.g. RCT) that are eligible for inclusion in the review. The preferred format includes both inclusion and exclusion criteria. If there are no restrictions on the types of study, this should be stated.

Peer-reviewed original qualitative research studies and qualitative reviews.

23. Context.

Give summary details of the setting or other relevant characteristics, which help define the inclusion or exclusion criteria.

24. * Main outcome(s).

Give the pre-specified main (most important) outcomes of the review, including details of how the outcome is defined and measured and when these measurement are made, if these are part of the review inclusion criteria.

Qualitative research findings, including from interview studies, focus group studies and ethnographic studies or other qualitative methods of data collection.

Measures of effect

25. * Additional outcome(s).

List the pre-specified additional outcomes of the review, with a similar level of detail to that required for main outcomes. Where there are no additional outcomes please state 'None' or 'Not applicable' as appropriate to the review

Not applicable

Measures of effect

26. * Data extraction (selection and coding).

Describe how studies will be selected for inclusion. State what data will be extracted or obtained. State how this will be done and recorded.

The research team will manually screen publications and identify those relevant for inclusion by screening articles by title and abstract. Duplicates will be removed and publications about which a member of the research team is uncertain will be flagged for a second member of the team to assess. Publications identified as relevant for inclusion will be imported into suitable software for analysis (e.g. NVivo or alternative).

27. * Risk of bias (quality) assessment.

State which characteristics of the studies will be assessed and/or any formal risk of bias/quality assessment tools that will be used.

Quality appraisal of the studies identified will be based on the Critical Skills Appraisal Checklist (CASP Checklist) qualitative studies checklist. Published studies will be assessed according to whether they address none, some or all of the CASP criteria. Articles will be independently appraised by more than one member of the research and judgements will be discussed to arrive at a consensus. Only studies that address some or all of the CASP criteria will be included in the synthesis although we will report on all of the studies that are included at the screening stage, including those that address none of the CASP checklist items. Qualitative reviews and evidence syntheses will be assessed using the Confidence in the Evidence from Reviews of Qualitative Research (GRADE-CERQual) approach to assess confidence in qualitative evidence synthesis (Lewin et al, 2018).

CASP Checklist: https://casp-uk.net/casp-tools-checklists/ [accessed 6th June 2022]

Lewin, S., Booth, A, Glenton, C. et al. Applying GRADE-CERQual to qualitative evidence synthesis findings: introduction to the series. Implementation Sci. 2018;13(2): https://doi.org/10.1186/s13012-017-0688-3

28. * Strategy for data synthesis.

Describe the methods you plan to use to synthesise data. This **must not be generic text** but should be **specific to your review** and describe how the proposed approach will be applied to your data.
If meta-analysis is planned, describe the models to be used, methods to explore statistical heterogeneity, and software package to be used.

The review will use a meta-synthesis approach to enable inclusion of studies that have used a range of qualitative data collection and analysis approaches. The studies will be analysed using a thematic analysis approach, in which material will be coded and grouped into themes that describe the relationship between social factors and pain transition. To ensure robust analysis, the research team will work together to double code and develop themes and categories on the studies.

The synthesis will be reported using the 'Enhancing transparency in the synthesis of qualitative research' statement (ENTREQ) (Tong et al, 2012).

Tong A, Flemming K, McInnes E, et al. Enhancing transparency in reporting the synthesis of qualitative research: ENTREQ. BMC Med Res Methodol 2012, 12(1):181). https://PubMed.ncbi.nlm.nih.gov/23185978/

29. * Analysis of subgroups or subsets.

State any planned investigation of 'subgroups'. Be clear and specific about which type of study or participant will be included in each group or covariate investigated. State the planned analytic approach.

No subgroups are planned at present, however, as some categories may be worthy of further analysis due to the detail, depth or breadth of their focus or findings, there may be further subgroup analyses if needed. These will use a similarly thematic approach.

30. * Type and method of review.

Select the type of review, review method and health area from the lists below.

Type of review

Cost effectiveness	No
Diagnostic	No
Epidemiologic	No
Individual patient data (IPD) meta-analysis	No
Intervention	No
Living systematic review	No
Meta-analysis	No
Methodology	No
Narrative synthesis	No
Network meta-analysis	No
Pre-clinical	No
Prevention	No
Prognostic	No
Prospective meta-analysis (PMA)	No
Review of reviews	No
Service delivery	No
Synthesis of qualitative studies	Yes
Systematic review	Yes
Other	No

Health area of the review

Alcohol/substance misuse/abuse	No
Blood and immune system	No
Cancer	No
Cardiovascular	No
Care of the elderly	No
Child health	No
Complementary therapies	No
COVID-19	No
Crime and justice	No

Dental	No
Digestive system	No
Ear, nose and throat	No
Education	No
Endocrine and metabolic disorders	No
Eye disorders	No
General interest	No
Genetics	No
Health inequalities/health equity	No
Infections and infestations	No
International development	No
Mental health and behavioural conditions	No
Musculoskeletal	No
Neurological	No
Nursing	No
Obstetrics and gynaecology	No
Oral health	No
Palliative care	No
Perioperative care	No
Physiotherapy	No
Pregnancy and childbirth	No
Public health (including social determinants of health)	Yes
Rehabilitation	No
Respiratory disorders	No
Service delivery	No
Skin disorders	No
Social care	No
Surgery	No
Tropical Medicine	No

Urological	No
Wounds, injuries and accidents	No
Violence and abuse	No

31. Language.

Select each language individually to add it to the list below, use the bin icon to remove any added in error.

English

There is not an English language summary

32. * Country.

Select the country in which the review is being carried out. For multi-national collaborations select all the countries involved.

England

Scotland

33. Other registration details.

Name any other organisation where the systematic review title or protocol is registered (e.g. Campbell, or The Joanna Briggs Institute) together with any unique identification number assigned by them.
If extracted data will be stored and made available through a repository such as the Systematic Review Data Repository (SRDR), details and a link should be included here. If none, leave blank.

34. Reference and/or URL for published protocol.

If the protocol for this review is published provide details (authors, title and journal details, preferably in Vancouver format)

No I do not make this file publicly available until the review is complete

35. Dissemination plans.

Do you intend to publish the review on completion?

Yes

We expect to produce peer reviewed publication/s.

36. Keywords.

Give words or phrases that best describe the review. Separate keywords with a semicolon or new line. Keywords help PROSPERO users find your review (keywords do not appear in the public record but are included in searches). Be as specific and precise as possible. Avoid acronyms and abbreviations unless these are in wide use.

pain; qualitative; chronic pain; persistent pain

37. Details of any existing review of the same topic by the same authors.

If you are registering an update of an existing review give details of the earlier versions and include a full bibliographic reference, if available.

38. * Current review status.

Update review status when the review is completed and when it is published.
New registrations must be ongoing so this field is not editable for initial submission.

Review_Ongoing

39. Any additional information.

Provide any other information relevant to the registration of this review.

Patient partners will be involved at key stages of the review. We will use an appropriate framework to guide Patient and Public Involvement and Engagement reporting.

40. Details of final report/publication(s) or preprints if available.

Leave empty until publication details are available OR you have a link to a preprint (NOTE: this field is not editable for initial submission).
List authors, title and journal details preferably in Vancouver format.
