# Supplementary material for: Social influences in the experience of transition to or from long-term (chronic) pain: A systematic review of qualitative research studies
Source: PLoS One. 2025 Jul 10;20(7):e0327984. doi: 10.1371/journal.pone.0327984 (PMC12244478; doi:10.1371/journal.pone.0327984)
Supplement: S2 File — (DOCX) [file pone.0327984.s002.docx]

**Supporting information file 2: Search strategy**

| Study Aim | The aim of this review was to synthesis qualitative research evidence to characterise how research about experiences has addressed interrelationships between social phenomena and pain, with focus on transitions to or from chronic pain. |
| --- | --- |
| Inclusion and exclusion | **Inclusion:** Studies published from 1979 – February 2025; Studies using empirical qualitative methods including interviews, focus groups, ethnographic or other qualitative methods of data collection, and reviews of qualitative studies; Qualitative studies focused on experience of transition to or from chronic pain; Studies including people aged 18 years and over; Studies in any language for which translation support is available  **Exclusion:** Quantitative studies in which the main method of data collection is a survey in which participants were asked to provide ‘free text’ responses to one or several survey questions only |
| Electronic data sources | EMBASE; PsycINFO; PubMed; CINAHL; Business Source Complete; Web of Science (including Social Sciences Citation Index); Scopus; Sociological Abstracts; Sociology Database |
| EMBASE | Title: ((Pain) OR (fibromyalgia) OR (headache disorders) OR (neuralgia) OR (neuropath) OR (osteoarthritis) OR (arthriti*))  Abstract: AND ((qualitative) OR (interview*) OR (focus group) OR (ethnograph*) OR (thematic analys*))  Abstract: AND ((age) OR (sex) OR (gender) OR (ethnic*) OR (educat*) OR (qualif*) OR (socioeconomic) OR (socio-economic) OR (depriv*) OR (rural) OR (urban) OR (employ*) OR (work) OR (occupation) OR (social) OR (support) OR (isolation) OR (lonel*) OR (marital status) OR (living arrangements) OR (living conditions) OR (organisational culture) OR (organizational culture) OR (household income) OR (financial difficulties) OR (leisure) OR (cultur*) OR (school) OR (communit*) OR (entrepreneur*))  Abstract: AND ((UK) OR (United Kingdom) OR (Great Britain) OR (British) OR (Engl*) OR (Wales) OR (Welsh) OR (Scotland) OR (Scottish) OR (Northern Ireland) OR (Northern Irish)) |
| PsychINFO | Title: ((Pain) OR (fibromyalgia) OR (headache disorders) OR (neuralgia) OR (neuropath) OR (osteoarthritis) OR (arthriti*))  Abstract: AND ((qualitative) OR (interview*) OR (focus group) OR (ethnograph*) OR (thematic analys*))  Abstract: AND ((age) OR (sex) OR (gender) OR (ethnic*) OR (educat*) OR (qualif*) OR (socioeconomic) OR (socio-economic) OR (depriv*) OR (rural) OR (urban) OR (employ*) OR (work) OR (occupation) OR (social) OR (support) OR (isolation) OR (lonel*) OR (marital status) OR (living arrangements) OR (living conditions) OR (organisational culture) OR (organizational culture) OR (household income) OR (financial difficulties) OR (leisure) OR (cultur*) OR (school) OR (communit*) OR (entrepreneur*))  Abstract: AND ((UK) OR (United Kingdom) OR (Great Britain) OR (British) OR (Engl*) OR (Wales) OR (Welsh) OR (Scotland) OR (Scottish) OR (Northern Ireland) OR (Northern Irish)) |
| PubMed | ((Pain[MeSH Terms]) OR (Pain[Title]) OR (fibromyalgia[MeSH Terms]) OR (headache disorder[MeSH Terms]) OR (neuralgia[Text Word]) OR (neuropath*[Text Word]) OR (osteoarthritis[Text Word]) OR (arthriti*[Text Word]))  AND ((qualitative[Title/Abstract]) OR (qualitative research[MeSH Terms]) OR (interview*[Text Word]) OR (focus group[Text Word]) OR (ethnograph*[Text Word]) OR (thematic analys*[Text Word]))  AND ((age[Text Word]) OR (sex[Text Word]) OR (gender[Text Word]) OR (ethnic*[Text Word]) OR (educat*[Text Word]) OR (qualif*[Text Word]) OR (socioeconomic[Text Word]) OR (socio-economic[Text Word]) OR (depriv*[Text Word]) OR (rural[Text Word]) OR (urban[Text Word]) OR (employ*[Text Word]) OR (work[Text Word]) OR (occupation[Text Word]) OR (social[Text Word]) OR (support[Text Word]) OR (isolation[Text Word]) OR (lonl*[Text Word]) OR (marital status[Text Word]) OR (living arrangements[Text Word]) OR (living conditions[Text Word]) OR (household income[Text Word]) OR (financial difficulties[Text Word]) OR (leisure[Text Word]) OR (cultur*[Text Word]) OR (school[Text Word]) OR (communit*[Text Word]) OR (entrepreneur[Text Word]))  AND ((UK[Text Word]) OR (United Kingdom[Text Word]) OR (Great Britain[Text Word]) OR (British[Text Word]) OR (Engl*[ Text Word]) OR (Wales[Text Word]) OR (Welsh[Text Word]) OR (Scotland[Text Word]) OR (Scottish[Text Word]) OR (Northern Ireland[Text Word]) OR (Northern Irish[Text Word])) |
| CINAHL | Title: ((Pain) OR (fibromyalgia) OR (headache disorders) OR (neuralgia) OR (neuropath) OR (osteoarthritis) OR (arthriti*))  Abstract: AND ((qualitative) OR (interview*) OR (focus group) OR (ethnograph*) OR (thematic analys*))  Abstract: AND ((age) OR (sex) OR (gender) OR (ethnic*) OR (educat*) OR (qualif*) OR (socioeconomic) OR (socio-economic) OR (depriv*) OR (rural) OR (urban) OR (employ*) OR (work) OR (occupation) OR (social) OR (support) OR (isolation) OR (lonel*) OR (“marital status”) OR (“living arrangements”) OR (“living conditions”) OR (“organisational culture”) OR (“organizational culture”) OR (“household income”) OR (“financial difficulties”) OR (leisure) OR (cultur*) OR (school) OR (communit*) OR (entrepreneur*))  Abstract: AND ((UK) OR (“United Kingdom”) OR (“Great Britain”) OR (British) OR (Engl*) OR (Wales) OR (Welsh) OR (Scotland) OR (Scottish) OR (“Northern Ireland”) OR (“Northern Irish”)) |
| Business Source Complete | Title: ((Pain) OR (fibromyalgia) OR (headache disorders) OR (neuralgia) OR (neuropath) OR (osteoarthritis) OR (arthriti*))  Abstract: AND ((qualitative) OR (interview*) OR (focus group) OR (ethnograph*) OR (thematic analys*))  Abstract: AND ((age) OR (sex) OR (gender) OR (ethnic*) OR (educat*) OR (qualif*) OR (socioeconomic) OR (socio-economic) OR (depriv*) OR (rural) OR (urban) OR (employ*) OR (work) OR (occupation) OR (social) OR (support) OR (isolation) OR (lonel*) OR (“marital status”) OR (“living arrangements”) OR (“living conditions”) OR (“organisational culture”) OR (“organizational culture”) OR (“household income”) OR (“financial difficulties”) OR (leisure) OR (cultur*) OR (school) OR (communit*) OR (entrepreneur*))  Abstract: AND ((UK) OR (“United Kingdom”) OR (“Great Britain”) OR (British) OR (Engl*) OR (Wales) OR (Welsh) OR (Scotland) OR (Scottish) OR (“Northern Ireland”) OR (“Northern Irish”)) |
| Web of Science (including Social Sciences Citation Index) | Title: ((Pain) OR (fibromyalgia) OR (headache disorders) OR (neuralgia) OR (neuropath) OR (osteoarthritis) OR (arthriti*))  Abstract: AND ((qualitative) OR (interview*) OR (focus group) OR (ethnograph*) OR (thematic analys*))  Abstract: AND ((age) OR (sex) OR (gender) OR (ethnic*) OR (educat*) OR (qualif*) OR (socioeconomic) OR (socio-economic) OR (depriv*) OR (rural) OR (urban) OR (employ*) OR (work) OR (occupation) OR (social) OR (support) OR (isolation) OR (lonel*) OR (“marital status”) OR (“living arrangements”) OR (“living conditions”) OR (“organisational culture”) OR (“organizational culture”) OR (“household income”) OR (“financial difficulties”) OR (leisure) OR (cultur*) OR (school) OR (communit*) OR (entrepreneur*))  Abstract: AND ((UK) OR (“United Kingdom”) OR (“Great Britain”) OR (British) OR (Engl*) OR (Wales) OR (Welsh) OR (Scotland) OR (Scottish) OR (“Northern Ireland”) OR (“Northern Irish”)) |
| Scopus | Article title, abstract, keywords, or author: ((Pain) OR (fibromyalgia) OR (headache disorders) OR (neuralgia) OR (neuropath*) OR (osteoarthritis) OR (arthriti*))  AND  Article title, abstract, keywords, or author: ((qualitative) OR (interview*) OR (focus group) OR (ethnograph*) OR (thematic analys*))  AND  Article title, abstract, keywords, or author: ((age) OR (sex) OR (gender) OR (ethnic*) OR (educat*) OR (qualif*) OR (socioeconomic) OR (socio-economic) OR (depriv*) OR (rural) OR (urban) OR (employ*) OR (work) OR (occupation) OR (social) OR (support) OR (isolation) OR (lonel*) OR (“marital status”) OR (“living arrangements”) OR (“living conditions”) OR (“household income”) OR (“financial difficulties”) OR (leisure) OR (cultur*) OR (school) OR (communit*) OR (entrepreneur*))  AND  Article title, abstract, keywords, or author: ((UK) OR (“United Kingdom”) OR (“Great Britain”) OR (British) OR (Engl*) OR (Wales) OR (Welsh) OR (Scotland) OR (Scottish) OR (“Northern Ireland”) OR (“Northern Irish”)) |
| Sociological Abstracts | Anywhere except full text: ((Pain) OR (fibromyalgia) OR (headache disorders) OR (neuralgia) OR (neuropath) OR (osteoarthritis) OR (arthriti*))  Anywhere except full text: AND ((qualitative) OR (interview*) OR (focus group) OR (ethnograph*) OR (thematic analys*))  Anywhere except full text: AND ((age) OR (sex) OR (gender) OR (ethnic*) OR (educat*) OR (qualif*) OR (socioeconomic) OR (socio-economic) OR (depriv*) OR (rural) OR (urban) OR (employ*) OR (work) OR (occupation) OR (social) OR (support) OR (isolation) OR (lonel*) OR (“marital status”) OR (“living arrangements”) OR (“living conditions”) OR (“organisational culture”) OR (“organizational culture”) OR (“household income”) OR (“financial difficulties”) OR (leisure) OR (cultur*) OR (school) OR (communit*) OR (entrepreneur*))  Anywhere except full text: AND ((UK) OR (“United Kingdom”) OR (“Great Britain”) OR (British) OR (Engl*) OR (Wales) OR (Welsh) OR (Scotland) OR (Scottish) OR (“Northern Ireland”) OR (“Northern Irish”)) |
| Sociology Database | Anywhere except full text: ((Pain) OR (fibromyalgia) OR (headache disorders) OR (neuralgia) OR (neuropath) OR (osteoarthritis) OR (arthriti*))  Anywhere except full text: AND ((qualitative) OR (interview*) OR (focus group) OR (ethnograph*) OR (thematic analys*))  Anywhere except full text: AND ((age) OR (sex) OR (gender) OR (ethnic*) OR (educat*) OR (qualif*) OR (socioeconomic) OR (socio-economic) OR (depriv*) OR (rural) OR (urban) OR (employ*) OR (work) OR (occupation) OR (social) OR (support) OR (isolation) OR (lonel*) OR (“marital status”) OR (“living arrangements”) OR (“living conditions”) OR (“organisational culture”) OR (“organizational culture”) OR (“household income”) OR (“financial difficulties”) OR (leisure) OR (cultur*) OR (school) OR (communit*) OR (entrepreneur*))  Anywhere except full text: AND ((UK) OR (“United Kingdom”) OR (“Great Britain”) OR (British) OR (Engl*) OR (Wales) OR (Welsh) OR (Scotland) OR (Scottish) OR (“Northern Ireland”) OR (“Northern Irish”)) |
