# Supplementary material for: Social influences in the experience of transition to or from long-term (chronic) pain: A systematic review of qualitative research studies
Source: PLoS One. 2025 Jul 10;20(7):e0327984. doi: 10.1371/journal.pone.0327984 (PMC12244478; doi:10.1371/journal.pone.0327984)
Supplement: S3 File — (DOCX) [file pone.0327984.s003.docx]

**Supporting information file 3: Critical Appraisal Skills Programme Checklist**

|  | Author, Year | Was there a clear statement of the aims of the research? | Is a qualitative methodology appropriate? | Was the research design appropriate to address the aims of the research? | Was the recruitment strategy appropriate to the aims of the research? | Was the data collected in a way that addressed the research issue? | Has the relationship between researcher and participants been adequately considered? | Have ethical issues been taken into consideration? | Was the data analysis sufficiently rigorous? | Is there a clear statement of findings? | How valuable is the research? |
| --- | --- | --- | --- | --- | --- | --- | --- | --- | --- | --- | --- |
| 1 | Ashe, et al. 2017 | y | Y | Y | Y | Y | CT | Y | Y | Y | Y |
| 2 | Ballard, et al. 2006 | y | Y | Y | Y | Y | CT | Y | Y | Y | Y |
| 3 | Belam, et al. 2005 | Y | Y | Y | Y | Y | Y | Y | Y | Y | Y |
| 4 | Bendelow and Williams 1996 | Y | Y | Y | Y | Y | CT | CT | CT | Y | Y |
| 5 | Brown, 2022 | Y | Y | Y | Y | Y | Y | Y | Y | Y | Y |
| 6 | Bruger, et al. 2023 | Y | Y | Y | Y | Y | Y | Y | Y | Y | Y |
| 7 | Chisholm, et al. 2016 | Y | Y | Y | Y | Y | CT | Y | Y | Y | Y |
| 8 | Clarke, et al. 2012 | Y | Y | Y | Y | Y | Y | Y | Y | Y | Y |
| 9 | Cole, et al. 2021 | Y | Y | Y | Y | Y | Y | Y | Y | Y | Y |
| 10 | Corbett, et al. 2007 | Y | Y | Y | Y | Y | Y | CT | Y | Y | Y |
| 11 | Crichton, and Wellock 2008 | Y | Y | Y | Y | Y | CT | Y | Y | Y | Y |
| 12 | Dennis, et al. 2013 | Y | Y | Y | Y | Y | Y | Y | Y | Y | Y |
| 13 | De Souza, and Frank 2011 | Y | Y | Y | Y | Y | Y | Y | Y | Y | Y |
| 14 | De Souza and Frank 2007 | Y | Y | Y | Y | Y | Y | Y | Y | Y | Y |
| 15 | Dibley et al. 2021 | Y | Y | Y | Y | Y | Y | Y | Y | Y | Y |
| 16 | Firth, et al. 2011 | Y | Y | Y | Y | Y | Y | Y | Y | Y | Y |
| 17 | Flurey, et al. 2017 | Y | Y | Y | Y | Y | Y | Y | Y | Y | Y |
| 18 | Flurey, et al. 2014 | Y | Y | Y | Y | Y | Y | Y | Y | Y | Y |
| 19 | Flurey, et al. 2018 | Y | Y | Y | Y | Y | Y | Y | Y | Y | Y |
| 20 | Gooberman-Hill, et al. 2017 | Y | Y | Y | Y | Y | Y | Y | Y | Y | Y |
| 21 | Grime et al. 2010 | Y | Y | Y | Y | Y | Y | Y | Y | Y | Y |
| 22 | Hadi, et al. 2019 | Y | Y | Y | Y | Y | Y | CT | Y | Y | Y |
| 23 | Hammond, et al. 2014 | Y | Y | Y | Y | Y | Y | Y | Y | Y | Y |
| 24 | Hearn, et al. 2015 | Y | Y | Y | Y | Y | Y | Y | Y | Y | Y |
| 25 | Holden, et al. 2012 | Y | Y | Y | Y | Y | Y | Y | Y | Y | Y |
| 26 | Holland and Collins, 2018 | Y | Y | Y | Y | Y | Y | Y | Y | Y | Y |
| 27 | Holloway, et al. 2000 | Y | Y | Y | Y | Y | Y | Y | CT | Y | Y |
| 28 | Hughes, 2009 | Y | Y | Y | Y | Y | Y | Y | Y | Y | Y |
| 29 | Jebara, et al. 2023 | Y | Y | Y | Y | Y | Y | Y | Y | Y | Y |
| 30 | Johnson, et al. 2016 | Y | Y | Y | Y | Y | Y | Y | Y | Y | Y |
| 31 | Johnson, et al. 2014 | Y | Y | Y | Y | Y | Y | Y | Y | Y | Y |
| 32 | Kett, et al. 2010 | Y | Y | Y | Y | Y | CT | Y | Y | Y | Y |
| 33 | Kinghorn, et al. 2015 | Y | Y | Y | Y | Y | CT | Y | Y | Y | Y |
| 34 | Kingstone, et al. 2020 | Y | Y | Y | Y | Y | Y | Y | Y | Y | Y |
| 35 | Leiper, et al. 2006 | Y | Y | Y | Y | Y | Y | Y | Y | Y | Y |
| 36 | Lempp, et al. 2006 | Y | Y | Y | Y | Y | CT | Y | Y | Y | Y |
| 37 | Mackichan, et al. 2013 | Y | Y | Y | Y | Y | Y | Y | Y | Y | Y |
| 38 | MacKichan, et al. 2013 | Y | Y | Y | Y | Y | CT | Y | Y | Y | Y |
| 39 | McMahon et al. 2012 | Y | Y | Y | Y | Y | Y | Y | Y | Y | Y |
| 40 | McParland, et al. 2011 | Y | Y | Y | Y | Y | Y | Y | Y | Y | Y |
| 41 | Maricar, et al. 2024 | Y | Y | Y | Y | Y | Y | Y | Y | Y | Y |
| 42 | Meehan, 2023 | Y | Y | Y | Y | Y | Y | Y | Y | Y | Y |
| 43 | Miles et al. 2005 | Y | Y | Y | Y | Y | Y | Y | Y | Y | Y |
| 44 | Morden, et al. 2011 | Y | Y | Y | Y | Y | Y | Y | Y | Y | Y |
| 45 | Morden, et al. 2017 | Y | Y | Y | Y | Y | Y | Y | Y | Y | Y |
| 46 | Morden, et al. 2015 | Y | Y | Y | Y | Y | Y | Y | Y | Y | Y |
| 47 | Osborn, et al. 1998 | Y | Y | Y | Y | Y | Y | Y | Y | Y | Y |
| 48 | Reynolds, et al. 2011 | Y | Y | Y | Y | Y | Y | Y | Y | Y | Y |
| 49 | Richardson, et al. 2014 | Y | Y | Y | Y | Y | Y | Y | Y | Y | Y |
| 50 | Richardson, et al. 2006 | Y | Y | Y | Y | Y | CT | Y | Y | Y | Y |
| 51 | Richardson, et al. 2007 | Y | Y | Y | Y | Y | CT | Y | Y | Y | Y |
| 52 | Robinson, et al. 2013 | Y | Y | Y | Y | Y | Y | Y | Y | Y | Y |
| 53 | Ryan and Roberts, 2019 | Y | Y | Y | Y | Y | Y | Y | Y | Y | Y |
| 54 | Sanders, et al. 2002 | Y | Y | Y | Y | Y | Y | Y | Y | Y | Y |
| 55 | Sanderson, et al. 2015 | Y | Y | Y | Y | Y | Y | Y | Y | Y | Y |
| 56 | Serbic and Pincus, 2013 | Y | Y | Y | Y | Y | Y | Y | Y | Y | Y |
| 57 | Singh, et al. 2018 | Y | Y | Y | Y | Y | Y | Y | Y | Y | Y |
| 58 | Smith, et al. 2018 | Y | Y | Y | Y | Y | Y | Y | Y | Y | Y |
| 59 | Smith and Osborn, 2007 | Y | Y | Y | Y | Y | CT | Y | Y | Y | Y |
| 60 | Snelgrove and Liossi, 2009 | Y | Y | Y | Y | Y | Y | Y | Y | Y | Y |
| 61 | Sofaer-Bennett, et al. 2007 | Y | Y | Y | Y | Y | Y | Y | Y | Y | Y |
| 62 | Sofaer, et al. 2005 | Y | Y | Y | Y | Y | Y | Y | Y | Y | Y |
| 63 | Taverner, et al. 2014 | Y | Y | Y | Y | Y | Y | Y | Y | Y | Y |
| 64 | Toye, et al. 2006 | Y | Y | Y | Y | Y | Y | Y | Y | Y | Y |
| 65 | Turner, et al. 2002 | Y | Y | Y | Y | Y | Y | Y | Y | Y | Y |
| 66 | Twiddy, et al. 2017 | Y | Y | Y | Y | Y | Y | Y | Y | Y | Y |
| 67 | Wainwright, et al. 2013 | Y | Y | Y | Y | Y | Y | Y | Y | Y | Y |
| 68 | Walker, et al. 1999 | Y | Y | Y | Y | Y | CT | Y | Y | Y | Y |
| 69 | Walker J, et al. 2006 | Y | Y | Y | Y | Y | Y | Y | Y | Y | Y |
| 70 | Watkins et al. 2020 | Y | Y | Y | Y | Y | Y | Y | Y | Y | Y |
| 71 | Wood, et al. 2017 | Y | Y | Y | Y | Y | Y | Y | Y | Y | Y |
