# Supplementary material for: Social influences in the experience of transition to or from long-term (chronic) pain: A systematic review of qualitative research studies
Source: PLoS One. 2025 Jul 10;20(7):e0327984. doi: 10.1371/journal.pone.0327984 (PMC12244478; doi:10.1371/journal.pone.0327984)
Supplement: S5 File — (DOCX) [file pone.0327984.s005.docx]

**Supporting information file 5: Extraction table**

|  | Author, Year | Pain Type/Condition | Pain duration | Sample (n) | Age range | Female (n) | Male (n) | Ethnicity | Analytical approach and Methods |
| --- | --- | --- | --- | --- | --- | --- | --- | --- | --- |
| 1 | Ashe, et al. 2017 | Fibromyalgia syndrome (FMS) | All participants had received official medical diagnoses | 14 | 29-58 | 12 | 2 | UK Citizens | Interpretative Phenomenological Analysis; Semi-structured in-depth interviews |
| 2 | Ballard, et al. 2006 | Pelvic pain | experienced pelvic pain for a median of 15 years (IQR 8–19.5 years) | 32 | 16-47 (median 32) | 32 | 0 | not reported | Thematic Analysis; Semi-structured interviews |
| 3 | Belam, et al. 2005 | Migraine | [duration not reported] participants attended the local headache clinic and had received a diagnosis of migraine | 8 | 30-61 | 6 | 2 | not reported | Participatory research/ Grounded theory; Trio interview: 2 researcher -1 participant interview |
| 4 | Bendelow and Williams 1996 | unclear | less than 1 year - 20+ | 34 | 20-80 | 22 | 12 | 2 men and 4 women - either Pakistani, Iranian or Greek Cypriot | Pilot Case study; Semi-structured interviews |
| 5 | Brown, 2022 | Fibromyalgia syndrome (FMS) | 3-10 years | 28 | not reported | 26 | 2 | not reported | Participatory, interpretative approach; Thematic Analysis; Completion identity box (art-based) and semi-structured online interviews |
| 6 | Bruger, et al. 2023 | Stroke survivors who experience chronic post-stroke pain | >3 months | 8 | 46-64 | 4 | 4 | White British | Interpretative phenomenological analysis; Semi-structured interviews |
| 7 | Chisholm, et al. 2016 | Psoriatic Arthritis | 4 months- 29 years | 24 | 27-71 | 11 | 13 | 21 White British; 3 Asian | Framework and thematic analysis - constant comparison technique; Semi-structured interviews |
| 8 | Clarke, et al. 2012 | Non-cancer chronic pain | 5-20 years | 23 | 65-89 | 16 | 7 | 16 Caucasian; 7 Chinese | Framework analysis; 2 x qualitative interviews (14/23 interviewed twice); group interview with Chinese participants |
| 9 | Cole, et al. 2021 | Endometriosis | 1.5-30 years | 34 | 22-56 | 34 | 0 | 30 white British or other European; 1 white and black Caribbean; 1 Asian; 1 Persian; 1 black/black British African | Feminist framework; reflexive Thematic Analysis; Qualitative online survey |
| 10 | Corbett, et al. 2007 | Chronic lower back pain | 12 weeks | 6 | 19-59 | 3 | 3 | not reported | Qualitative component of a mixed method study; Interviews |
| 11 | Crichton, and Wellock 2008 | Symphysis pubis dysfunction (SPD); Pelvic girdle pain (PGP) | Unclear, they define what is meant by chronic pain in the intro but no specific sample details in methodology | 28 | 18-24 | 28 | 0 | No specifics: The cultural mix of the women was varied as was the socio-economic status, which was in keeping with the geographical area | Qualitative phenomenological approach; Semi-structured interviews. 3 interviewed on 3 occasions; 17 interviewed twice; 8 interviewed once |
| 12 | Dennis, et al. 2013 | Fibromyalgia | 1-15 years | 20 | 18-64 | 20 | 0 |  | Hermeneutic phenomenological approach; Email semi-structured interviews |
| 13 | De Souza, and Frank 2011 | Chronic low back pain (CLBP) | 6 months - 29 years | 11 | 27-79 | 6 | 5 | 6 Indian; 1 Mauritian; 1 Sikh; 1 English; 1 French; 1 Irish | Qualitative Framework approach; Thematic content analysis ; In-depth interviews (topics rather than questions were used as a guide) |
| 14 | De Souza and Frank 2007 | Chronic back pain (CBP) | 6 months - 29 years | 11 | 27-79 | 6 | 5 | 6 Indian; 1 Mauritian; 1 Sikh; 1 English; 1 French; 1 Irish | Framework approach; thematic content analysis; Unstructured interviews |
| 15 | Dibley et al. 2021 | Inflammatory bowel disease (IBD) | Disease duration 2-40 years | 26 | 21-60 | 15 | 11 | 63% White/White British, 7.5% were Asian/Asian British, 7.5% were Black/Black British, 7.5% were of mixed ethnicity, and 14.5% did not specify their ethnic group. | exploratory qualitative research (EQR); Framework analysis guided by Common Sense Model of Illness Representations; 7 focus groups; fieldnotes |
| 16 | Firth, et al. 2011 | Foot ulceration with Rheumatoid Arthritis | Rheumatoid Arthritis: 5-64 years; ulcer duration 2-104 weeks | 23 | 45-88 | 17 | 6 | not reported | Thematic framework; In-depth interviews |
| 17 | Flurey, et al. 2017 | Rheumatoid Arthritis | 5-21 years | 22 | 44-75 | 0 | 22 | All white British | Thematic Analysis; 6 focus groups |
| 18 | Flurey, et al. 2014 | Rheumatoid Arthritis | 3-30 years | 15 | 35-77 | 12 | 3 | not reported | Inductive Thematic Analysis; Semi-structured interviews |
| 19 | Flurey, et al. 2018 | Rheumatoid Arthritis | 2-19 years | 5 | 49-69 | 0 | 22 | All white British | Case study; Thematic Analysis; Semi-structured interviews |
| 20 | Gooberman-Hill, et al. 2017 | Knee and hip pain | <1 year | 28 | 57-89 | 14 | 14 | not reported | Questerviews; analysis constant comparison; 6 focus groups |
| 21 | Grime et al. 2010 | Osteoarthritis; pain in peripheral joints | include only those who had long-standing peripheral joint pain/osteoarthritis of at least medium intensity, the baseline interview revealed that responders reported a range of conditions | 27 | 56-87 | 15 | 12 | not reported | Qualitative longitudinal study; Thematic Analysis; in-depth interviews using life grid approach (family, work, leisure, and health over life course, present and future). Followed by 11 month of diaries sheets to pick up changes |
| 22 | Hadi, et al. 2019 | pain type/condition reported for the quant - qual not specifically separated | >3 months | 19 | 27-74 | 8 | 11 | not reported | A convergent parallel mixed-methods design; Thematic Analysis; Semi structured interviews |
| 23 | Hammond, et al. 2014 | Chronic sports injury | The duration of these injuries ranged from playing in two matches with an acute injury to managing injuries over an entire competitive season (up to 10 months or more) | 9 | mean age 28.7 ± 3.39 years | 0 | 9 | not reported | Coding matrix; Content analysis; Semi-structured existential-phenomenological interviews |
| 24 | Hearn, et al. 2015 | Spinal cord injury | min 3 months | 8 | 26-77 | 3 | 5 | not reported | Interpretative Phenomenological Analysis; Semi-structured interviews |
| 25 | Holden, et al. 2012 | Knee pain | Over 3 months - within the last year | 21 | 50-80 | 11 | 10 | not reported | Mixed methods. Qual analysis: inductive constant comparison method; Semi-structured interviews |
| 26 | Holland and Collins, 2018 | Rheumatoid Arthritis | 1-15 years | 11 | 32-58 | 9 | 2 | All white British | Thematic Analysis; in-depth semi-structured interviews |
| 27 | Holloway, et al. 2000 | Chronic back pain (CBP) | 2-52 years | 18 | 28-80 | not reported | not reported | not reported | Report in different places that they conduct Thematic analysis AND Narrative analysis; in-depth Unstructured interviews |
| 28 | Hughes, 2009 | Rheumatoid Arthritis | 3 - 35 years | 13 | 51-72 | not reported | not reported | not reported | Grounded theory; Analysed using open, axial and selective coding; 2 unstructured interviews; 11 Semi-structured interviews |
| 29 | Jebara, et al. 2023 | Polymyalgia; Arthritis; Metastasised cancer | >12 weeks | 14 | 75-89 | 7 | 7 (3 gender not identified) | White | Thematic Analysis; semi-structured telephone interviews |
| 30 | Johnson, et al. 2016 | Knee pain prior to surgery | Exact pain duration not reported but going in for surgery so can assume chronic | 10 | 61-78 | 4 | 6 | not reported | Interpretative Phenomenological Analysis; Semi-structured and follow-up post-operative interviews |
| 31 | Johnson, et al. 2014 | Osteoarthritis; Hip replacement | Exact pain duration not reported but going in for surgery so can assume chronic | 23 | 52-82 | 11 | 13 | not reported | Inductive thematic analysis: Semi-structured and follow-up post-operative interviews |
| 32 | Kett, et al. 2010 | Rheumatoid Arthritis | 0.5-12 years (disease duration) | 21 | 23-72 | 14 | 7 | South Asian: 10; White:9; Afro-Caribbean:2 | Grounded theory approach; Semi-structured interviews |
| 33 | Kinghorn, et al. 2015 | Back pain; Fibromyalgia; arthritis; widespread pain | All patients, however, were experiencing pain which was sufficiently severe to justify referral to a specialist clinic. | 16 | 33-81 | 9 | 7 | not reported | Participatory methods; grounded approach; Focus group |
| 34 | Kingstone, et al. 2020 | osteoarthritis; Muscular pain; Sciatica; Post shingles | 3 months or more | 8 | mean: 78 years | 4 | 4 | All White British | Ethnographic observations, analysis informed by constructivist grounded theory; Semi-structured interviews; Data comprised 29 h of audio-recorded data from interviews, a fieldnote journal, and 101 photographs taken by participants (for the purposes of photo-elicitation interviews) or the researcher during go-along interviews or to supplement fieldnotes |
| 35 | Leiper, et al. 2006 | Headaches | 3-45 years | 17 | 36-65 | 8 | 9 | not reported | Framework approach utilising thematic analysis; Semi-structured interviews |
| 36 | Lempp, et al. 2006 | Rheumatoid Arthritis | 1-29 years | 26 | Mean:56 | 22 | 4 | African, 1; Black, 1; British/English/White, 17; Caribbean, 3; Chinese–Mauritian, 1; Jamaican, 1; West Indian, 1; White other, 1 | Content analysis; Semi-structured interviews |
| 37 | Mackichan, et al. 2013 | Osteoarthritis; spondylosis; Arthritis; Worn cartilage; Damaged discs; Polymyalgia rheumatica; Sciatica; Fibromyalgia; Angina; rheumatoid arthritis; Disc degeneration; Tendonitis; Crohns disease; Ligament and muscle damage | 1-45 years | 31 | 67-92 | 16 | 15 | not reported | Mixed methods. Qualitative: Constructive grounded theory; Semi-structured interviews. Life grid |
| 38 | MacKichan, et al. 2013 | Back Pain | Back pain in the preceding 6 months | 23 | 38-78 | not reported | not reported | not reported | Mixed methods. Qualitative: Constant comparative method; Semi structured interviews |
| 39 | McMahon et al. 2012 | Fibromyalgia | 18 months - 15 years | 10 | 25-70 | 10 | 0 | white British | Narrative inquiry and analysis; Semi-structured interviews |
| 40 | McParland, et al. 2011 | Participants reported upper (e.g. head, neck) (n = 3, 20.0%), middle (e.g. hips, abdomen) (n = 5, 33.3%), lower (e.g. knees, ankles) (n = 5, 33.3%) and all over body pain (n = 2, 13.3%). | at least 6 months | 15 | 18-65 | 8 | 7 | not reported | Interpretative Phenomenological Analysis; 3 focus groups; semi-structured interviews |
| 41 | Maricar, et al. 2024 | Chronic Widespread pian; fibromyalgia; osteoarthritis; inflammatory arthritis | Physicians diagnosis associated with chronic pain | 12 | 40-68 | 9 | 3 | Pakistan; Indian; Bangladesh | Thematic Analysis; Semi-structured interviews |
| 42 | Meehan, 2023 | Spondylolisthesis; frozen shoulder; back and neck pain; pelvic pain; fibromyalgia; ME and fatigue | >3 months | 6 | 30-50 | 5 | 1 | not reported | Thematic Analysis; Semi-structured interviews |
| 43 | Miles et al. 2005 | primary pain sites: lower back or lower back and legs, 28% had pain located in the legs (not back), 21% mentioned more than two main sites of pain, 7% had head pain, 3% neck pain and 3% chest pain. Twenty-one percent said the cause of their pain was unknown and a further 7% described it as due to ‘wear and tear’. | Persisted for more than 3 months; 1-59 years | 29 | 31-84 | 20 | 9 | not reported | Grounded theory; Open ended interviews |
| 44 | Morden, et al. 2011 | Chronic knee pain | No reporting of pain duration - there are clues that the pain is chronic and pain is described as moderate/severe within the last year | 22 | 50-74 | 13 | 9 | not reported | Constant comparative method and narrative methods; in-depth baseline & follow-up interviews and diary |
| 45 | Morden, et al. 2017 | Chronic knee pain | no reporting of actual pain duration, just that they sufferer chronic pain - sampled based on self-completion of WOMAC | 22 | 56-90 | 13 | 9 | not reported | Elements of grounded theory; Semi-structured interviews, follow-up interviews and Diary |
| 46 | Morden, et al. 2015 | Chronic knee pain | No reporting of pain duration - there are clues that the pain is chronic -title refers to chronic joint pain moderate/severe pain within the last year, and throughout the pain is referred to as chronic | 22 | not reported | not reported | not reported | not reported | Elements of grounded theory; Diaries and 'follow-up' interviews |
| 47 | Osborn, et al. 1998 | Back pain | at least 5 years | 9 | 25-55 | 9 | 0 | not reported | Interpretative phenomenological analysis; Semi-structured interviews |
| 48 | Reynolds, et al. 2011 | Arthritis | 1-60 Years | 12 | 62-81 | 12 | 0 | All were White and currently lived in the UK, although two had previously lived in other European countries. | Interpretative Phenomenological Analysis; Semi-structured interviews |
| 49 | Richardson, et al. 2014 | Chronic joint pain (Osteoarthritis) | Those who reported no health problem and/or no limitation and/or mild pain were excluded. [no specific pain duration for sample is reported] Findings: As described above, we selected participants who indicated on cohort study questionnaires that their musculoskeletal condition was chronic and problematic | 21 | 55-90 | not reported | not reported | not reported | Thematic Analysis; in-depth interviews; life grid approach; 11months of diary sheets |
| 50 | Richardson, et al. 2006 | Chronic widespread pain | Lasted for at least 3 months | 8 | 40-58 | 4 | 4 | not reported | Interpretative Phenomenological Analysis; life grid interviews, diaries and diary interviews |
| 51 | Richardson, et al. 2007 | Chronic widespread pain | lasted for at least three months | 8 | 40-60 | 4 | 4 | not reported | Analysis was a combination of the thematic and the narrative; Serial in-depth interviews, including life grid; unstructured diary interviews |
| 52 | Robinson, et al. 2013 | Rheumatoid arthritis; Back pain and/or fibromyalgia; Slipping rib syndrome; Rheumatoid arthritis and spinal tuberculosis | 48-144 months | 5 | 25-78 | 4 | 1 | not reported | Discourse analysis; In-depth lightly structured interviews |
| 53 | Ryan and Roberts, 2019 | Leg pain; Back pain; Foot pain; | 3-month to 9-year | 14 | 34-81 | 8 | 6 | not reported | Interpretative Phenomenological Analysis; Semi-structured interviews |
| 54 | Sanders, et al. 2002 | Osteoarthritis of the hip and/or knee | Sampled based on New Zealand scores to indicate level of need for hip and knee replacement surgery based on pain, disability and clinical assessment | 27 | 51-91 | 17 | 10 | not reported | Constant comparison; Semi-structured in-depth interviews |
| 55 | Sanderson, et al. 2015 | Rheumatoid Arthritis | <1 - 35 | 16 | 29-62 | 16 | 0 | Punjabi | Grounded theory approach; Semi-structured interviews |
| 56 | Serbic and Pincus, 2013 | Chronic low back pain (CLBP) | 1-10+ years | 20 | Age mean and SD: 46.4 (14.7) | 14 | 6 | not reported | Grounded theory approach; Semi-structured interviews |
| 57 | Singh, et al. 2018 | Chronic low back pain | 1-25 years | 10 | 23-53 | 5 | 5 | 5 English-speaking Punjabi and 5 white British | Thematic Analysis; Semi- structured interviews |
| 58 | Smith, et al. 2018 | Patellofemoral pain | 3 months to 16 years | 10 | 26-37 | 7 | 3 | not reported | Thematic Analysis; Semi-structured interviews |
| 59 | Smith and Osborn, 2007 | Chronic benign low back pain | 5-15 years | 6 | 36-52 | 2 | 4 | European, Caucasian, | Interpretative Phenomenological Analysis; Semi-structured interviews |
| 60 | Snelgrove and Liossi, 2009 | Chronic low back pain | 4 years | 10 | 39-66 | 7 | 3 | Caucasian | Interpretative Phenomenological Analysis; Semi-structured interviews |
| 61 | Sofaer-Bennett, et al. 2007 | Spinal 60.3% Knee 7.9% Shoulder 6.5% Post surgery/trauma 7.9% Other (chest, headaches, facial, abdominal, perineal) 18.0% | more than 3 months | 63 | 60-80+ | 42 | 20 | not reported | Grounded Theory; Constant comparison Unstructured, in-depth interviews |
| 62 | Sofaer, et al. 2005 | Spinal 60.3% Knee 7.9% Shoulder 6.5% Post surgery/trauma 7.9% Other (chest, headaches, facial, abdominal, perineal) 18.0% | more than 3 months | 63 | 60-80+ | 42 | 21 | not reported | Grounded Theory; Constant comparison Unstructured, in-depth interviews |
| 63 | Taverner, et al. 2014 | Chronic leg ulceration | 7 months - 20 years | 11 | 65> | 9 | 2 | white | Grounded theory; Semi-structured interviews |
| 64 | Toye, et al. 2006 | Knee osteoarthritis (OA) | The WOMAC pain scores for those interviewed ranged from 5–50 (median 35), and WOMAC function scores ranged from 6–51 (median 41). | 18 | 60-76 | 6 | 12 | not reported | Interpretative Phenomenological Analysis; Semi-structured interviews |
| 65 | Turner, et al. 2002 | Osteoarthritis | 1-46 years | 12 | 45-76 | 0 | 12 | not reported | Interpretative Phenomenological Analysis; Semi-structured telephone interviews |
| 66 | Twiddy, et al. 2017 | Growing pains [in the title, no reporting of other pain conditions] | Reported as: chronic pain condition [but no specific reporting on pain duration - all attended a pain clinic - FG's over 4 month period] | 18 | 18-30 | 15 | 3 | not reported | Constant comparison analysis; Focus group |
| 67 | Wainwright, et al. 2013 | Fibromyalgia (5) – Back (4) – Joint hyper mobility syndrome (2) – Osteoarthritis (2) – Sciatica (2); Neck (2); Hip (1); Knee (1); Spine (1); Undiagnosed general (1) | Pain lasting over 3 months within the last year | 13 | not reported | 5 | 8 | not reported | Constructivist grounded theory principles; Semi-structured interviews |
| 68 | Walker, et al. 1999 | Chronic low back pain | 2-50 years | 20 | 28-80 | 8 | 12 | not reported | Thematic Analysis; Unstructured interviews |
| 69 | Walker J, et al. 2006 | Chronic back pain | The median duration of pain was eight years with a range of two to ﬁfty years | 20 | 28-79 | 8 | 12 | British and white | Interpretative Phenomenological Analysis; unstructured interviews/narrative approach |
| 70 | Watkins et al. 2020 | Knee injury: anterior cruciate ligament (ACL) rupture | 1-10 years | 12 | 19-35 | 6 | 6 | not reported | Thematic Analysis; Semi-structured interviews |
| 71 | Wood, et al. 2017 | Chronic prostatitis/chronic pelvic pain | 4 - 31 (years) | 12 | 34– 42 years | 0 | 12 | not reported | Narrative thematic analysis; Semi-structured interview (7 face2Face; 2 online) |

Name of data extractors: Information about study characteristics and methods were extracted by the first author (SS) in collaboration with co-authors (EW, AG, CG) using an electronic spreadsheet so that key information was extracted accurately and consistently.

Date of data extraction: October 2022 / Extraction of the search update February 2025
