# Supplementary material for: Social influences in the experience of transition to or from long-term (chronic) pain: A systematic review of qualitative research studies
Source: PLoS One. 2025 Jul 10;20(7):e0327984. doi: 10.1371/journal.pone.0327984 (PMC12244478; doi:10.1371/journal.pone.0327984)
Supplement: S6 File — (DOCX) [file pone.0327984.s006.docx]

**Supporting information file 6: Thematic map**

| Theme | Sub theme | Codes |
| --- | --- | --- |
| The role of social connections with family, friends and community | The role of family | Social networks |
|  | The role of friendships | Friends |
|  | Social connections within the local community | Neighbours and wider community members |
|  | The role of social isolation | Caring for others |
|  |  | Restricted interactions with social network |
|  |  | Trade and services |
|  |  | Lack of or avoiding support |
|  |  | Losing social circles |
|  |  | Isolation or loneliness or separation |
|  |  | Interaction with support network |
|  |  | Family burden |
|  |  | Family demand |
|  |  | Changes to relational dynamics |
|  |  | Intergenerational dynamics |
|  |  | Parenting with pain |
|  |  | Grandchildren |
|  |  | Self-care and daily functioning |
|  |  | Volunteering |
|  |  | Local community |
|  |  | Public transport |
| The role of lifestyles | Daily routine household tasks | Lifestyle |
|  | Commensality and nutrition | Nutrition |
|  | Sociality of sleep | Sleep |
|  | Participating in social activities | Household and chores |
|  | Participating in hobbies | Shopping and daily tasks |
|  |  | Social activities and social lives |
|  |  | Socialising and participation |
|  |  | Exercise and sport |
|  |  | Leisure and hobbies |
|  |  | Special occasions |
|  |  | Withdrawal, restrictions and less active |
|  |  | Weather and seasonal changes |
| Occupation | Workplace relations | Work related pain |
|  | Retirement | Autonomy in the workplace |
|  | Financial disadvantage | Supportive employers |
|  |  | Unsupportive employers |
|  |  | Changes to work pattern |
|  |  | Needing and wanting to work |
|  |  | Workplace adjustments |
|  |  | Retirement |
|  |  | Issues around disclosure |
|  |  | Absence and sickness |
|  |  | Job loss and unsuitability of employment |
|  |  | Organisation structure |
|  |  | Presenteeism |
|  |  | Colleagues |
|  |  | Benefits |
|  |  | Financial constraints |
|  |  | Education |
