# Supplementary material for: Social influences in the experience of transition to or from long-term (chronic) pain: A systematic review of qualitative research studies
Source: PLoS One. 2025 Jul 10;20(7):e0327984. doi: 10.1371/journal.pone.0327984 (PMC12244478; doi:10.1371/journal.pone.0327984)
Supplement: S7 File — (DOCX) [file pone.0327984.s007.docx]

**Supporting information file 7:** **Themes and exemplar quotes from primary studies**

| Sub themes | Thematic sentence to describe a pain context or transition [primary studies supporting evidence] | Example of quotations to support thematic sentence, including page numbers and [primary studies supporting evidence] |
| --- | --- | --- |
| The role of the family | People push through the pain, and ignore it in as much as is possible to participate in family activities or social events, accepting that some activities will have future consequences for their pain worsening [34, 36, 65, 72, 74]. | ‘Julie: I usually just get on with things and take anti-inﬂammatories to dull the pain if it happens when I have a social event. I tend to be good at covering up if it is affecting me and suffering in silence, so people wouldn’t usually know there is a problem’ p.184 [34]; ‘I’ve always danced. My wife and I did ballroom dancing. I struggled at times but then you just go through the pain barrier and try to ignore it. (Lewis, late 70s)’ p.601 [72]; ‘Today I am very tired and in quite a lot of pain, I went ﬁshing with my grandson for the full day yesterday. Even though I enjoy the sport and have warm gear and wet gear, it still knocks me about….’ p.193 [74]. |
| The role of friendship | Friends try their best to understand but still expect a lot from the friendship or forget due to the invisibility of the pain condition [36, 41, 58]. | ‘They do their best to understand, however, they expect a lot of me, regardless. (Ann)’ p.774 [36]; ‘I don’t make a big point of it. Er a couple of me friends know about it’. He explained that this was because most people either ‘don’t understand anyway’ or forget, due to the invisible nature of the condition: ‘There’s nothing about you that looks different. Even close friends forget about it at times.’ Mark also avoids talking about his RA due to the lack of understanding from friends, who make vicious jokes’ p.118 [41]. |
| Social connections and the local community | Pain can be exacerbated when visiting family and friends due to negotiating different designs of environments, crossing roads and using public transport to access the local community [13, 54, 59, 90]. | ‘You need a bit more room all the time to cross and you look and you’ve got to sort of say to yourself you’re not going do that. You might end up in a heap in the middle of the road. At least if you end up in a heap in the middle of the road on a zebra crossing, they’re all stopped. You going to, you’re not going to get run over. Michael, 2nd interview’ p.897 [13]; ‘Extract from ﬁeldnotes: The cobbles of the car park were raised and seemed unhelpful for somebody using a wheeled walker. The early morning rain had left a slippery top to the stones and the runnels between them made pushing three wheels suﬃciently tricky’ p.133 [15]; ‘Mary for example, recently stopped using public transport, and no longer visited friends’ p.704 [90]. |
| The role of social isolation | Studies reported the tension between needing to withdraw from social life and the fear of feeling isolated [12, 34, 35] | ‘But I mean we just don’t go, we won’t go anywhere now because of that I get too embarrassed and I just hate being in company and you always get onto that subject [pain]. And if you’re out for social evening the last thing people want to hear is what your misery is, so I just, that’s why we don’t go out that often (Becky)’ p.74 [12]; ‘But I already feel like I have put enough strain on my friends and family … I suffer in as much silence as I can muster and carry on. Endo has got to be the most lonely condition I can imagine’ p.184 [34]. |
| Daily routine and household tasks | For example, pushing and pulling a vacuum cleaner [35], standing for long period to cook [46], completing household repairs [54, 66], and participating in daily household tasks was often reported as difficult and painful, which in some cases resulted in relying on others to complete everyday tasks [11, 35, 46, 47, 49, 50, 61, 78] | ‘Nearly all the women in the Gap study mentioned that pushing and pulling the vacuum cleaner was very diﬃcult and painful. As a consequence, the task of vacuuming was neglected, or it had to wait until someone else could do it for her’ p.11 [35]; ‘… at times is very painful because if I do cook for up to half and hour in the kitchen, I have to lay down, to put my legs up’ p.592 [46]; ‘I get so frustrated sometimes I have broken down and cried because I can’t even drill, I can’t decorate, I can’t do anything’ pp. 201-202 [54]; ‘Some women participants report that their spouses had taken on household tasks such as vacuum cleaning, formerly performed by the women’ p.380 [61]. |
| Commensality and nutrition | Where it is possible to manage the diet within the limits of living with others, some foods are avoided or increased when believed to influence the experience of pain [36, 48, 49]. | ‘I identiﬁed some triggers which I have avoided for years, such as caffeine, alcohol, foods with additive…Even now I develop sensitivities to foods I eat too often so have to vary my diet (as much as I can within limits)’ p.770 [36]; ‘For example, South Asian participants were more likely to alter their diet to control their ﬂares, and described following a more traditional diet, containing herbs such as fennel, ginger and garlic (Table 7, quotation 8), and avoiding greasy food’ p.209 [49]. |
| Sociality of sleep | It is reported that sleep disruption occurs from sharing a bed with a partner [35] and when there is limited support, women sleep on the sofa, sometimes for extended periods of time and sometimes with their children [35, 50]. | ‘One woman admitted that she spent a week in the same clothes because she could not use the stairs and had no help that week. In this instance, mother and child slept on the settee’ p.9 [35]; 'Finally sorted out a bed in the spare room so I don't have to sleep with my husband ... I need all the room ... because every time I turn over, it's a big ordeal ... I wake have up and then if I did get comfortable and their he moved and made me move ... rye would have a big row and it was hard to get to sleep' (VM11)’ p.10 [35]; ‘I lived on the settee for 7 months; I couldn’t get up the stairs. I couldn’t sleep through pain, it was awful. We had a really difficult patch and I had to explain to my boyfriend that when I am in pain my sexual drive is gone. (Female patient 14, White other, 42 years, RA for 1–5 years)’ p.50 [50]. |
| Participation in social activities | Studies reported the difficulties of attending celebratory events (e.g., weddings, funerals, parties, socialising events) due to not being able to accommodate their pain, which can result in family upset or pushing through the pain, which makes their pain worse in the days that follow [12, 36, 66, 69, 86]. | ‘I didn’t even go out Christmas or New Year because I knew what it would be like, there’d be no sitting down because it would be all packed and there’s no way I’d like to stand up and if there were a seat I’d have to get back up so I can’t remember the last time we went out’ p.74 [12]; ‘Special occasions, such as weddings, meals out and parties were described as a ‘nightmare’ (female; 6 months)’ p.1406 [66]; ‘I mean like I love amusement rides and stuff like that you know once you get that adrenalin rush … you just get so excited especially when you’ve got the kids beside you. You know and by the end of the day you know, you know you’ve done too much, you know that you shouldn’t have done it and you get up the following morning and you get really angry with yourself, you know, and then you get rebellious because you think to yourself hey fuck it you know’ p.438 [86]. |
| Participating in hobbies | Enjoyment was reported as a reason to maintain the meaningful activities, pushing through the pain, knowing that their pain may increase as a result [65, 72, 73, 77]. | ‘I want to go white-water rafting I’ll go white-water rafting and believe me I have. I did pay for it the next day but you know, I enjoyed it at the time. (Participant 3, F, 37)’ p.699 [65]; ‘I do realise that I’m potentially doing myself some harm by continuing to play with all the risks factors that I have – so it’s got to be worth it, it’s got to be fun (YA004)’ P.6 [73]; ‘I play golf and of course, doing the twisting that you do, I do feel it on occasion, but I can’t allow it to stop me doing that, because I enjoy it (413)’ p.1559 [77]. |
| The role of occupation | Studies reported that people want to continue working and value the stimulation that work provides, along with social interaction, sense of purpose and joy, income, a goal to aim for, to feel proud and influence the way others perceive them and use work as a distraction, where the pain is still present but feels more bearable [17, 41, 55, 59, 74]. | ‘Work was highly valued and the “need” to work commonly expressed: the need to work to be productive; to earn; to keep physically and mentally active; to gain social stimulation; and to provide distraction from pain and fatigue’ p.407 [17]; ‘Well, I use work a lot, I find it really helps to get so distracted. It doesn't go away exactly but somehow it's more bearable ... (male, self-employed architect, aged 45)’ p.1132 [55]; ‘The pain from his knee caused him diﬃculties when bending and also prevented him from placing his knee on hard surfaces for too long. Michael works as a manual labourer for a large supermarket chain. In his account, he discussed the importance of having to work to help keep the family home, arguably part of a moral need to maintain a sense of competency in his social role’ p.194 [74]. |
| Workplace relationships | The type of work, working environment, length of hours worked are reported to influence the pain experience [19, 41]. | ‘After six months I got really bad so I had to stop for good. It was all factory work ... We used to stand in our job nearly all day. My legs used to give way (Jai: ﬁrst, None/L)’ p.2329 [19]; ‘Both Mark and David seem to have enough autonomy in their roles that they can manage to work around their RA. Whilst David has control over his hours and the tasks he undertakes (‘I can plan my day’), Mark is self-employed and alters his work to accommodate his RA: I am getting into more servicing of boilers and maintenance, because obviously the installs are getting harder and harder’ pp.120-121 [41]. |
| Retirement | For some, early retirement increased social isolation and changed the dynamics of their relationships [16, 74], whereas others reported that retirement gave them more time to cope with the pain [41]. | ‘Some perceived that early retirement from work on grounds of ill-health had increased their social isolation and altered relationships with their husbands. However, others celebrated the new directions that their lives had taken since retiring from work’ p.330 [16]; ‘The exercise is very important to me but it’s everything that goes along with it. I’ve made new friends since I retired and I just ﬁnd that completely satisfying; I enjoy it and I don’t want to give that up’ Catherine, 2nd interview’ p.195 [74]; ‘Charles argued that it is easier to deal with RA when retired: ‘not having to work gives you the time to cope’ p.124 [41]. |
| Financial disadvantage | Job loss or reduced hours signalled the loss of financial independence, reduced social lives, support, social status and role shifts within family dynamics [35, 42, 47, 50, 54, 71, 88]. | ‘Some felt that their ability to help support the family had been compromised by the condition of SPD and this aﬀected how they felt about themselves’ p.8 [35]; ‘I mean I worked for nearly 40 years, there is a certain loss of identity when you can’t work anymore ... When you can’t work, not only have you taken away your sort of daily structure, you have taken away a large part of your social life. (Female patient 4, English, 57 years, RA for 6–11 years)’ p.115 [50]; ‘Job loss was reported by all of the participants of working age and appeared to rank high in terms of life disruption because of its domino eﬀect in precipitating other losses’ p.202 [54]. |
