# Supplementary material for: Social influences in the experience of transition to or from long-term (chronic) pain: A systematic review of qualitative research studies
Source: PLoS One. 2025 Jul 10;20(7):e0327984. doi: 10.1371/journal.pone.0327984 (PMC12244478; doi:10.1371/journal.pone.0327984)
Supplement: Attachment — Submitted filename: Response to Reviewers.docx [file pone.0327984.s009.docx]

Manuscript ID: PONE-D-25-11915

30^th^ May 2025

**Re: resubmission of manuscript, ‘Social influences in the experience of transition to or from long-term (chronic) pain: a systematic review of qualitative research studies.’**

Dear Editor of *PLOS ONE,*

Thank you for the careful review of our qualitative systematic review manuscript. We were grateful for the constructive comments that have enabled us to revise the manuscript. We believe that the revisions made substantially improve the submission by enhancing clarity. Below we respond to each point raised by the academic Editors and Reviewers, which we reproduce in bold italics.

The revisions have been developed in consultation with all co-authors, and each author has given approval to the final form of this revision.

Yours sincerely

Samantha Stone, PhD *and on behalf of the authors*

Bristol Medical School,

University of Bristol, BS8 1PN, UK

**Table of revision tasks to meet journal requirements**

| ***Requirements*** | | ***Author response/actions*** |
| --- | --- | --- |
| ***1.*** | ***Please ensure that your manuscript meets PLOS ONE's style requirements, including those for file naming. The PLOS ONE style templates can be found at***  [***https://journals.plos.org/plosone/s/file?id=wjVg/PLOSOne_formatting_sample_main_body.pdf***](https://track.editorialmanager.com/CL0/https:%2F%2Fjournals.plos.org%2Fplosone%2Fs%2Ffile%3Fid=wjVg%2FPLOSOne_formatting_sample_main_body.pdf/1/010f01966d058c44-94955af5-662a-42c8-a969-72eec2de7b51-000000/QQfvRPCaFg22LxCePKvX-JoRYxR1uACCumnBu4LJbx4=208)  ***and***  [***https://journals.plos.org/plosone/s/file?id=ba62/PLOSOne_formatting_sample_title_authors_affiliations.pdf***](https://track.editorialmanager.com/CL0/https:%2F%2Fjournals.plos.org%2Fplosone%2Fs%2Ffile%3Fid=ba62%2FPLOSOne_formatting_sample_title_authors_affiliations.pdf/1/010f01966d058c44-94955af5-662a-42c8-a969-72eec2de7b51-000000/psLndx2Kg-udh4MioTSDFzwxaPD-PMtyINdyqFnX2vo=208) | - Following **manuscript body formatting guidance**, we have added an acknowledgement section to the end of the manuscript (p.33), this is in addition to previously following **title, author, affiliation formatting guidance** requires acknowledgements to be added after author information (p.1) |
| ***2.*** | ***Please ensure that your PRISMA flow diagram is included in your main manuscript file as Figure 1; please see the PLOS ONE submission guidelines for systematic reviews and meta-analyses at*** [***https://journals.plos.org/plosone/s/submission-guidelines#loc-systematic-reviews-and-meta-analyses***](https://journals.plos.org/plosone/s/submission-guidelines#loc-systematic-reviews-and-meta-analyses)***.*** | - Prisma flow diagram added to the manuscript (p.10) - Reference to the review protocol has been moved from the introduction/objectives section to the beginning of the method section (p.7) |
| ***3.*** | ***Please include your tables as part of your main manuscript and remove the individual files. Please note that supplementary tables (should remain/ be uploaded) as separate "supporting information" files*** | - Table 1. inclusion and exclusion has been added (p.7) - Due to readability and flow of the manuscript we have added Table 2. Themes and exemplar quotes from primary studies to a supporting information file (S6 file). We added information to that effect on page 14. |
| ***4.*** | ***Please include captions for your Supporting Information files at the end of your manuscript, and update any in-text citations to match accordingly. Please see our Supporting Information guidelines for more information:*** [***http://journals.plos.org/plosone/s/supporting-information***](http://journals.plos.org/plosone/s/supporting-information)***.*** | - Captions for supporting information files have been added to the end of the manuscript (p.40), in-text citations match accordingly (p.8; p.9; p.11; p.13; p.14; p.30). |
| ***5.*** | ***As required by our policy on Data Availability, please ensure your manuscript or supplementary information includes the following:***  ***A numbered table of all studies identified in the literature search, including those that were excluded from the analyses.***  ***For every excluded study, the table should list the reason(s) for exclusion.***  ***If any of the included studies are unpublished, include a link (URL) to the primary source or detailed information about how the content can be accessed.***  ***A table of all data extracted from the primary research sources for the systematic review and/or meta-analysis. The table must include the following information for each study:***  ***Name of data extractors and date of data extraction***  ***Confirmation that the study was eligible to be included in the review.***  ***All data extracted from each study for the reported systematic review and/or meta-analysis that would be needed to replicate your analyses.***  ***If data or supporting information were obtained from another source (e.g. correspondence with the author of the original research article), please provide the source of data and dates on which the data/information were obtained by your research group.***  ***If applicable for your analysis, a table showing the completed risk of bias and quality/certainty assessments for each study or outcome.  Please ensure this is provided for each domain or parameter assessed. For example, if you used the Cochrane risk-of-bias tool for randomized trials, provide answers to each of the signalling questions for each study. If you used GRADE to assess certainty of evidence, provide judgements about each of the quality of evidence factor. This should be provided for each outcome.***  ***An explanation of how missing data were handled.***  ***This information can be included in the main text, supplementary information, or relevant data repository. Please note that providing these underlying data is a requirement for publication in this journal, and if these data are not provided your manuscript might be rejected.*** | - A column and numbers added to the table for all studies (S3 and S5 files). No studies were excluded from the analyse. - All included studies are published - Information about data extractors and how the studies eligibility was confirmed is within the manuscript (p.13). Name of the extractors and dates of extraction have been added to the end of S5 File. - No data was obtained from another sources - Quality assessment for each study is included in S3 file. Quality was assessed using CASP, which is the appropriate method for assessing qualitative research. - As this is a thematic synthesis of qualitative research evidence. There is no missing data that we need to handle. This item is not applicable to the approach taken. - In addition, we have updated S4 file. ENTREQ statement, so xref elements match the manuscript |
| ***6.*** | ***Please review your reference list to ensure that it is complete and correct. If you have cited papers that have been retracted, please include the rationale for doing so in the manuscript text, or remove these references and replace them with relevant current references. Any changes to the reference list should be mentioned in the rebuttal letter that accompanies your revised manuscript. If you need to cite a retracted article, indicate the article’s retracted status in the References list and also include a citation and full reference for the retraction notice.*** | - To our knowledge, none of the references that have been included have been retracted since their publication. |

**Table of tasks to address Reviewers’ comments**

| **reviewer #1** | ***Author response/actions*** |
| --- | --- |
| ***Dear Authors,  I am pleased to review your manuscript entitled "Social Influences in the Experience of Transition to or from Long-Term (Chronic) Pain: A Systematic Review of Qualitative Research Studies." The manuscript is well-written, comprehensive, and contributes meaningfully to the literature on chronic pain and social influences.*** | Thank you for the careful reading of our submission, we were pleased to learn that you found the manuscript well-written, comprehensive and a valuable contribution to the chronic pain literature. Your encouraging comments were greatly appreciated by our team, thank you. |
| ***However, I have a few suggestions to further strengthen the quality of the paper:  Abstract – Objective Statement: In the objectives section of the abstract, please revise the phrase “we aimed” to the more academically appropriate form “this study aimed.” This change will enhance the formal tone of the manuscript in line with scholarly writing conventions.*** | Thank you for the useful comment. As suggested, we have removed the words ‘we aimed’ to a more formal academic tone ‘this study aimed’ (p.2). Thank you. |
| ***Methods Section – Trustworthiness and Saturation: While the methodology is generally robust, I recommend including a subsection or explicit content under the methods section addressing how trustworthiness and data saturation were assessed or discussed in the included studies. These are essential elements in evaluating qualitative research rigor. For guidance, I suggest integrating insights and references from the following recent publications:***  [***https://doi.org/10.1016/j.glmedi.2024.100171***](https://doi.org/10.1016/j.glmedi.2024.100171)  [***https://doi.org/10.1016/j.glmedi.2024.100051***](https://doi.org/10.1016/j.glmedi.2024.100051)  [***https://doi.org/10.1016/j.glmedi.2025.100198***](https://doi.org/10.1016/j.glmedi.2025.100198) | Thank you. We agree that addressing trustworthiness and data saturation is crucial for evaluating the rigour of qualitative research. In response, we have added content to the discussion within the ‘Study quality appraisal’ section (p.11). Here we have added description of how we examined the included studies for evidence of methodological rigour. Drawing on the CASP criteria, we have added more detailed description of on how we assessed saturation or data adequacy in the studies that were included in the review. |
| ***Reviewer #2*** | ***Author response/actions*** |
| ***Consider more directly emphasizing how future research can overcome methodological limitations (e.g., prospective qualitative work on individuals transitioning out of pain).*** | Thank you for this thoughtful suggestion. We have revised the discussion section to emphasise how future research can address methodological limitations in the current evidence base. In particular, we now highlight the need for prospective qualitative studies that follow individuals transitioning into and out of pain, as this approach could provide deeper insight into change processes and mechanisms that are difficult to capture retrospectively. The revised content can be found on pages 29-30. |
| ***Some conceptual definitions, such as “transition,” could benefit from clearer operationalization.*** | Thank you. We have added to the definition of ‘transition’ in the revised manuscript, specifically how it is operationalised within the context of this review. The change appears in the introduction (p.5) and additions to the discussion offer further clarity to how we are operationalising the concept (pp.29-30). |
| ***The discussion might be enhanced by further comparing themes to relevant theoretical frameworks (e.g., social models of disability or identity theory).*** | Thank you for this helpful suggestion. We have strengthened the discussion by situating the review themes within relevant social sciences theories that illuminate how chronic pain can disrupt, reshape or reinforce aspects of identity, self-concept and social roles. We also draw on socio-ecological models to illustrate how themes interact and reinforce one another, underscoring the importance of social and wider contextual dimensions that impact on the experience of chronic pain. The revisions are located on pages 28-29. |
